# Association of diet and outdoor time with inflammatory bowel disease: a multicenter case-control study using propensity matching analysis in China

**DOI:** 10.3389/fpubh.2024.1368401

**Published:** 2024-06-17

**Authors:** Xiaotian Chu, Xuanfu Chen, Huimin Zhang, Yufang Wang, Hong Guo, Yan Chen, Xiaowei Liu, Zhenhua Zhu, Yao He, Xueli Ding, Qunying Wang, Changqing Zheng, Xiaocang Cao, Hong Yang, Jiaming Qian

**Affiliations:** ^1^Department of Gastroenterology, Peking Union Medical College Hospital, Chinese Academy of Medical Sciences & Peking Union Medical College, Beijing, China; ^2^Department of Gastroenterology, Sichuan University West China Hospital, Chengdu, China; ^3^Department of Gastroenterology, Xinqiao Hospital, Army Medical University, Chongqing, China; ^4^Department of Gastroenterology, The Second Affiliated Hospital, Zhejiang University School of Medicine, Hangzhou, China; ^5^Department of Gastroenterology, Xiangya Hospital, Central South University, Changsha, China; ^6^Department of Gastroenterology, The First Affiliated Hospital of Nanchang University, Nanchang, China; ^7^Department of Gastroenterology, The First Affiliated Hospital, Sun Yat-sen University, Guangzhou, China; ^8^Department of Gastroenterology, The Affiliated Hospital of Qingdao University, Qingdao, China; ^9^Gastroenterology and Hepatology, Affiliated Jinhua Hospital, Zhejiang University School of Medicine, Hangzhou, China; ^10^Department of Gastroenterology, Shengjing Hospital, China Medical University, Shenyang, China; ^11^Department of Gastroenterology and Hepatology, Tianjin Medical University General Hospital, Tianjin, China

**Keywords:** ulcerative colitis, Crohn's disease, environmental factors, outdoor time, case-control study

## Abstract

**Objective:**

To investigate the association between dietary and some other environmental factors and the risk of inflammatory bowel diseases (IBD) in Chinese population.

**Materials and methods:**

A multicenter case-control study was conducted involving 11 hospitals across China. A total of 1,230 subjects were enrolled consecutively, and diet and environmental factor questionnaires were collected. IBD patients were matched with healthy controls (HC) using propensity-score matching (PSM) at a 1:1 ratio with a caliper value of 0.02. Multivariate conditional logistic regression analyses were performed to evaluate the associations between diet, environmental factors, and IBD.

**Results:**

Moderate alcohol and milk consumption, as well as daily intake of fresh fruit, were protective factors for both Crohn's disease (CD) and ulcerative colitis (UC). Conversely, the consumption of eggs and chocolate increased the risk of IBD. Outdoor time for more than 25% of the day was a protective factor only for CD. In eastern regions of China, CD patients had higher egg consumption and less outdoor time, while UC patients consumed more chocolate. IBD patients from urban areas or with higher per capita monthly income consumed more fruit, eggs, and chocolate.

**Conclusions:**

This study reveals an association between specific foods, outdoor time, and the emergence of IBD in the Chinese population. The findings emphasize the importance of a balanced diet, sufficient outdoor time and activities, and tailored prevention strategies considering regional variations.

## 1 Introduction

Inflammatory bowel disease (IBD), including Crohn's disease (CD) and ulcerative colitis (UC), has traditionally been considered a high-prevalence disease in Western countries. However, it is now becoming a global disease in the 21st century (1). Recent studies have shown that the incidence of IBD has been on the rise since the end of the last century in newly industrialized regions or countries in South America, Africa, and Asia, which mirrors the pattern of IBD epidemiology observed in Western countries over 50 years ago during a stage of rapid socioeconomic development (1–3). For instance, in the past two decades, the incidence of IBD in Hong Kong has increased from 1.0 to 3.1 per 100,000 (4). Similarly, in Yunnan province, mainland China, the crude incidence of IBD has risen from 0.068 to 1.152 between 1998 and 2013 (5). Furthermore, the age-standardized prevalence of CD in China has increased from 1.59 to 3.39 per 100,000 between 2013 and 2016, while the prevalence of UC has risen from 8.72 to 17.24 per 100,000 during the same period (6). A joinpoint regression and age-period-cohort analysis published in 2022 has also demonstrated an increase in age-standardized incidence and a decrease in age-standardized mortality rates of IBD, which explained the rise in age-standardized prevalence in the last three decades (7). Given the country's large population and aging population, these findings indicate an increasing disease burden of IBD in China, which will be a major public health challenge in the near future. Therefore, it is crucial to explore potential risk factors for IBD in order to improve disease prevention and management.

The increasing incidence of IBD is believed to be influenced by environmental factors associated with urbanization, industrialization, economic development, and changes in lifestyle, particularly diet. The changes in diet patterns are reflected mainly in cooking methods as well as dietary structure after urbanization/industrialization. A traditional diet or diet of inhabitants from rural regions usually consists of more raw plant of fiber-based foods relative to a western diet, which is common in urban regions and loosely defined as one high in saturated fats, red meat and carbohydrates and low in fresh fruits, vegetables and whole grain (8). Easily acquired highly processed food is another characteristic of diet in urban regions. The shift in dietary patterns may contribute significantly to the development of IBD (9, 10). In an umbrella review of meta-analyses conducted by Piovani et al. (11), it was found that drinking tea can reduce the risk of UC. Similarly, Cui et al., in a systematic review, reported that heavy sugar intake, daily consumption of eggs, and frequent consumption of fried foods increase the risk of CD and UC while tea and bean intake have a protective effect against CD and UC in mainland China (12). Several other factors have also been found to be associated with IBD. For instance, smoking has been found to be a protective factor in UC but a risk factor in CD, whereas alcohol appears to be protective against UC but does not seem to have any influence on CD (10). Additionally, it has been suggested that undergoing an appendectomy or tonsillectomy may be a risk factor for CD (13).

Due to increasingly indoor lifestyles and potential sun protection measures, daily exposure to sunshine has decreased over time (14). Sunshine exposure is a crucial source of vitamin D. Research suggests that low levels of sunshine exposure, as well as vitamin D deficiency, are associated with an increased risk of IBD and may even contribute to disease exacerbations (15). The lifestyle characterized by reduced outdoor time and sunshine exposure may have an impact on the development of IBD, which deserves further attention.

The evolving epidemiology of IBD in China has recently shown an increasing trend, characterized by an acceleration in incidence (3). Therefore, it is of great necessity to identify the risk and protective factors associated with the onset of IBD in China. However, there have been relatively few studies conducted on the Chinese population regarding factors related to the development of IBD. Previous research has yielded inconsistent results regarding the role of alcohol, dairy products, and eggs in the emergence of IBD. To address these gaps, we conducted a multicenter case-control study with a large sample size, trying to explore the association between environmental factors and the development of IBD.

## 2 Materials and methods

### 2.1 Study population

This is a multicenter case-control study conducted across 11 hospitals in different cities in China. From January 1, 2018, to August 31, 2019, a total of 1,230 subjects who met the following criteria were consecutively enrolled. Individuals included in this research were from 27 provinces across China and the distribution of them was shown in Supplementary Table 1.

The inclusion criteria for the IBD group were as follows: (1) aged 18 years or older; (2) patients who met the clinical diagnosis criteria for CD or UC, in accordance with the Chinese and European Crohn's and Colitis Organization (ECCO) consensus on the diagnosis and treatment of IBD (16–18); (3) able to recall diet before IBD emergence; and (4) completion of the questionnaire.

Healthy controls (HC) were selected based on the following criteria: (1) family members of the IBD patients or individuals from the same region as the cases, matched for age and gender; (2) aged 18 years or older; (3) absence of chronic diseases such as IBD or IBD symptoms (e.g., abdominal pain, shapeless stool, frequent diarrhea, etc), other immune disorders, tumors, etc.; (4) no history of long-term medication use; (5) not more than one family members diagnosed IBD; and (6) completion of the questionnaire.

Subjects were excluded if they met any of the following criteria: (1) incomplete questionnaire or (2) failure to provide informed consent.

In the end, a total of 667 HCs were included in the study. All HCs were individuals from the same regions with the IBD patients, and some of them were parents, siblings, or children of the IBD patients. The protocols of this study were approved by the Peking Union Medical College Hospital and the Chinese Academy of Medical Sciences Ethics Committee (JS-347), as well as other participating medical centers. All research was conducted in accordance with relevant guidelines and regulations, and informed consent was obtained from all participants.

### 2.2 Questionnaire design

The diet questionnaire (Supplementary Table 2) was designed based on food frequency questionnaire (19), which is a reliable tool to collect diet information in large population. The questionnaire we used comprised seven participant information questions and 20 food intake questions related to various food items concerning diet patterns before IBD onset, such as fresh vegetables, fresh fruit, red meat, fresh fish, salted fish, shellfish, crabs, shrimps, milk, yogurt, eggs, Western-style fast food, fried food, coarse grain, tea, coffee, food stored in the refrigerator for more than 3 days, raw seafood, raw vegetables, and chocolate. Additionally, there were three questions about habits, including tobacco and alcohol intake, the percentage of daily outdoor time, as well as one question regarding past medical history, which included appendectomy, tonsillectomy, cholecystectomy, asthma, eczema, or Helicobacter pylori (HP) infection before the age of 20 and disease onset. The amount of food consumed per meal was collected using the questionnaire, and food items mentioned above, which were consumed in quantities exceeding 50 g per meal, were further counted and analyzed. Food and alcohol intake frequency were categorized into three levels: never (never consumed), occasionally (once a week to once a month), and every day (consumed daily). Smoking status was defined as either never smoked or ever smoked (including those who previously smoked but have now quit and those who currently smoke). The percentage of daily outdoor time was categorized as <25%, 25%−50%, or more than 50%. For patients with IBD, all survey contents referred to the situation prior to the onset of gastrointestinal symptoms.

### 2.3 Data collection methods

The researchers utilized standardized language and an electronic survey to collect basic information on diet, smoking, alcohol intake, and past medical history. Subsequently, two individuals independently analyzed and verified the data to assign values to the research variables and ensure accuracy.

### 2.4 Statistical analysis

IBD patients and HC were matched at a 1:1 ratio using propensity-score matching (PSM) with a caliper value of 0.02 on variables including age, gender, ethnic group (Han or ethnic minorities), family history of IBD, education level (universities and higher education or not), and birthplace (urban or suburban area). The PSM procedure aimed to minimize bias caused by confounding variables in the database, and the effectiveness of the matching was assessed using the standardized mean difference (SMD). Continuous variables were presented as mean ± standard deviation (SD) or median [interquartile range, (IQR)] based on their distribution. Differences between the matched groups were evaluated using Student's *t*-tests or Mann–Whitney *U*-tests as appropriate. Categorical data are presented as numbers (percentages), and differences between groups were examined using chi-square analysis. Subsequently, multivariate conditional logistic regression analyses were performed to assess the associations between environmental factors, past medical history, and the risk of UC or CD. The consumption levels of alcohol, fresh vegetables, red meat, fresh fish, salted fish, shellfish, crabs, shrimps, milk, yogurt, eggs, and coarse grain were measured using eating frequency and the amount of food consumed per meal were also collected through the questionnaire. The amount of food consumed per meal was estimated based on the average while the others was measured only by eating frequency. Subgroup analysis was conducted to explore dietary and regional variations in IBD patients and IBD patients were divided into different subgroups according to their birthplace (eastern regions, middle regions and western regions of China, urban and suburban regions) and different levels of per capita monthly income, respectively.

## 3 Results

### 3.1 The baseline characteristics of the study population

A total of 1,230 subjects were included in the study, including 377 CD patients, 186 UC patients, and 667 healthy controls. After PSM, 314 CD patients were successfully matched with 314 HC, and 164 UC patients were paired with 164 HC. The baseline characteristics of CD and UC patients compared to HC before and after PSM are presented in Supplementary Tables 3, 4, respectively. After PSM, the SMD for all variables decreased to 0.1 or less, indicating the success of the matching procedure, and the distribution of propensity score was shown in Supplementary Figures 1, 2, which illustrated the balance between patients and healthy controls. In comparison to the HC groups, there were no statistically significant differences in gender (male: 67.5% in HC vs. 67.8% in CD and 50.6% in HC vs. 50.6% in UC; *P* > 0.05). The median age was 35.00 (29.25–44.75) in HC and 34.00 (29.00–41.75) in CD, as well as 40.00 (32.75–50.00) in HC and 39.00 (33.00–49.00) in UC, respectively (*P* > 0.05). No statistically significant differences were observed in ethnic group, family history of IBD, education level, or birthplace between the groups (*P* > 0.05).

### 3.2 Questionnaire survey on factors associated with IBD onset

The questionnaire used to examine factors associated with IBD onset included assessments of diet, habits, and past medical history. Univariate analysis was conducted to compare the differences between IBD patients and HC (Supplementary Table 5).

#### 3.2.1 Questionnaire survey on factors associated with CD onset

CD patients exhibited a significantly higher percentage of never drinking alcohol (*P* < 0.001), never consuming fresh fruit (*P* = 0.001), consuming red meat occasionally or every day (*P* = 0.005), consuming fresh fish occasionally or every day (*P* = 0.001), never drinking milk (*P* < 0.001), consuming eggs occasionally or every day (*P* = 0.001), never consuming coarse grain (*P* < 0.001), never drinking tea (*P* = 0.005), never consuming raw seafood (*P* = 0.002), never consuming raw vegetables (*P* = 0.008), occasional consumption of chocolate (*P* = 0.045), and lower levels of outdoor time (*P* = 0.003) compared to HC (Supplementary Table 5).

In the multivariate analysis, occasional alcohol intake [vs. never, odds ratio (OR) (95% confidence interval, CI): 0.253 (0.067–0.485)], daily consumption of fresh fruit [vs. never, OR = 0.216 (0.075–0.625)], occasional consumption of milk [vs. never, OR = 0.174 (0.058–0.518)], and outdoor time for 25%−50% and over 50% of the day [occasionally vs. <25% of the day, OR = 0.363 (0.138–0.955); every day vs. <25% of the day, OR = 0.201 (0.050–0.807)] were identified as protective factors for CD (Table 1). On the other hand, consumption of eggs [occasionally vs. never, OR = 4.908 (1.891–12.739); every day vs. never, OR = 5.218 (1.858–14.657)] and occasional consumption of chocolate [vs. never, OR = 4.938 (1.840–13.249)] were identified as risk factors for CD onset. The average amounts of alcohol, milk, and eggs consumed per meal were (66.09 ± 64.90) g, (160.34 ± 100.73) g, and (86.33 ± 48.46) g, respectively. Smoking tended to be associated with a higher risk of CD, but the OR was 3.139 (0.982–10.037; *P* = 0.054).

**Table 1:** The risk and protective factors of Crohn's disease.

	**OR (95% CI)**	***P*-value**
**Smoking**		
Never	1.00 (ref)	
Ever	3.139(0.982–10.037)	0.054
**Alcohol intake**		
Never	1.00 (ref)	
Occasionally	0.253 (0.067–0.485)	0.001
Every day	–	0.979
**Fresh fruit**		
Never	1.00 (ref)	
Occasionally	0.844 (0.292–2.439)	0.753
Every day	0.216 (0.075–0.625)	0.005
**Milk**		
Never	1.00 (ref)	
Occasionally	0.174 (0.058–0.518)	0.002
Every day	0.413 (0.132–1.292)	0.129
**Egg**		
Never	1.00 (ref)	
Occasionally	4.908 (1.891–12.739)	0.001
Every day	5.218 (1.858–14.657)	0.002
**Chocolate**		
Never	1.00 (ref)	
Occasionally	4.938 (1.840–13.249)	0.002
Every day	8.983 (0.091–887.140)	0.349
**Outdoor time of a day**		
<25%	1.00 (ref)	
25–50%	0.363 (0.138–0.955)	0.040
>50%	0.201 (0.050–0.807)	0.024

OR, odds ratio; CI, confidence interval; ref, reference.

#### 3.2.2 Questionnaire survey on factors associated with UC onset

In the univariate analysis, higher percentages of UC patients were observed to never drink alcohol (*P* < 0.001), never eat fresh fruit or consume fresh fruit occasionally (*P* = 0.007), never drink milk (*P* = 0.006), consume eggs occasionally or every day (*P* = 0.017), never drink coffee or consume coffee occasionally (*P* = 0.023), never eat raw seafood (*P* = 0.016), never eat raw vegetables or consume raw vegetables occasionally (*P* = 0.049), and consume chocolate occasionally (*P* = 0.001; Supplementary Table 5).

In the multivariate analysis, alcohol intake [occasionally vs. never, OR = 0.222 (0.103–0.476); every day vs. never, OR = 0.038 (0.002–0.702)], daily consumption of fresh fruit [vs. never, OR = 0.411 (0.178–0.947)], and consumption of milk [occasionally vs. never, OR = 0.274 (0.130–0.579); every day vs. never, OR = 0.304 (0.099–0.935)] were identified as protective factors for UC. Conversely, consumption of eggs [occasionally vs. never, OR = 3.741 (1.647–8.495); every day vs. never, OR = 4.499 (1.931–10.484)] and occasional consumption of chocolate [vs. never, OR = 3.099 (1.395–6.885)] were identified as risk factors for UC (Table 2). The average amounts of alcohol, milk, and eggs consumed per meal were (70.24 ± 61.41) g, (177.53 ± 95.94) g, and (89.06 ± 47.33) g, respectively.

**Table 2:** The risk and protective factors of ulcerative colitis.

	**OR (95% CI)**	***P-*value**
**Alcohol intake**		
Never	1.00 (ref)	
Occasionally	0.222 (0.103–0.476)	<0.001
Every day	0.038(0.002–0.702)	0.028
**Fresh fruit**		
Never	1.00 (ref)	
Occasionally	1.147 (0.524–2.512)	0.732
Every day	0.411 (0.178–0.947)	0.037
**Milk**		
Never	1.00 (ref)	
Occasionally	0.274 (0.130–0.579)	0.001
Every day	0.304 (0.099–0.935)	0.038
**Egg**		
Never	1.00 (ref)	
Occasionally	3.741(1.647–8.495)	0.002
Every day	4.499 (1.931–10.484)	<0.001
**Chocolate**		
Never	1.00 (ref)	
Occasionally	3.099 (1.395–6.885)	0.005
Every day	–	0.990

OR, odds ratio; CI, confidence interval; ref, reference.

### 3.3 Subgroup analysis for dietary and regional variations in IBD patients

To further analyze the differences in diet among different populations of IBD patients, we conducted a subgroup analysis.

We observed that the percentage of CD patients who consumed eggs every day was highest in the eastern regions of China and lowest in the western regions (Table 3, *P* = 0.016). However, no significant difference in outdoor time was observed among CD patients in different regions (*P* > 0.05).

**Table 3:** The difference in diet and outdoor time among Crohn's disease patients from different regions in China.

	**Eastern regions *N* = 143**	**Middle regions *N* = 143**	**Western regions *N* = 91**	***P*-value**
**Alcohol intake [*****n*** **(%)]**				
Never	122 (85.3)	120 (83.9)	81 (89.0)	0.455
Occasionally	19 (13.3)	23 (16.1)	9 (9.9)	
Every day	2 (1.4)	0 (0.0)	1 (1.1)	
**Fresh fruit [*****n*** **(%)]**				
Never	45 (31.5)	33 (23.1)	17 (18.7)	0.144
Occasionally	56 (39.2)	72 (50.3)	45 (49.5)	
Every day	42 (29.4)	38 (26.6)	29 (31.9)	
**Milk [*****n*** **(%)]**				
Never	106 (74.1)	107 (74.8)	69 (75.8)	0.320
Occasionally	22 (15.4)	24 (16.8)	19 (20.9)	
Every day	15 (10.5)	12 (8.4)	3 (3.3)	
**Egg [*****n*** **(%)]**				
Never	34 (23.8)	31 (21.7)	27 (29.7)	0.016
Occasionally	56 (39.2)	68 (47.6)	49 (53.8)	
Every day	53 (37.1)	44 (30.8)	15 (16.5)	
**Chocolate [*****n*** **(%)]**				
Never	106 (74.1)	109 (76.2)	71 (78.0)	0.841
Occasionally	37 (25.9)	33 (23.1)	20 (22.0)	
Every day	0 (0.0)	1 (0.7)	0 (0.0)	
**Percentage of outdoor time [*****n*** **(%)]**				
<25%	69 (69.7)	64 (68.8)	35 (59.3)	0.426
25%−50%	26 (26.3)	25 (26.9)	18 (30.5)	
>50%	4 (4.0)	4 (4.3)	6 (10.2)	

Significant differences were observed in the diet and outdoor time of CD patients between suburban and urban regions (Table 4). The percentage of CD patients who consumed fresh fruit every day, eggs every day, milk, and chocolate was higher in urban regions. Additionally, urban CD patients had a higher percentage of <25% outdoor time.

**Table 4:** The difference in diet and outdoor time between Crohn's disease patients from suburban and urban regions in China.

	**Suburban regions *N* = 218**	**Urban regions *N* = 159**	***P*-value**
**Alcohol intake [*****n*** **(%)]**			
Never	189 (86.7)	134 (84.3)	0.720
Occasionally	27 (12.4)	24 (15.1)	
Every day	2 (0.9)	1 (0.6)	
**Fresh fruit [*****n*** **(%)]**			
Never	49 (22.5)	46 (28.9)	0.025
Occasionally	113 (51.8)	60 (37.7)	
Every day	56 (25.7)	53 (33.3)	
**Milk [*****n*** **(%)]**			
Never	174 (79.8)	108 (67.9)	0.007
Occasionally	34 (15.6)	31 (19.5)	
Every day	10 (4.6)	20 (12.6)	
**Egg [*****n*** **(%)]**			
Never	42 (19.3)	50 (31.4)	0.001
Occasionally	117 (53.7)	56 (35.2)	
Every day	59 (27.1)	53 (33.3)	
**Chocolate [*****n*** **(%)]**			
Never	180 (82.6)	106 (66.7)	<0.001
Occasionally	37 (17.0)	53 (33.3)	
Every day	1 (0.5)	0 (0.0)	
**Percentage of time outdoor time [*****n*** **(%)]**			
<25%	94 (61.0)	74 (76.3)	0.019
25%−50%	52 (33.8)	17 (17.5)	
>50%	8 (5.2)	6 (6.2)	

Furthermore, when considering per capita monthly income, CD patients with higher incomes had a higher percentage of occasional alcohol consumption, daily fresh fruit and egg consumption, as well as occasional chocolate intake (Table 5). However, they had a lower proportion of outdoor time.

**Table 5:** The difference in diet and outdoor time among Crohn's disease patients with different levels of per capita monthly income in China.

	** <2,000 yuan *N* = 73**	**2,000–5,000 yuan *N* = 185**	**5,000–10,000 yuan *N* = 87**	**>10,000 yuan *N* = 32**	***P-*value**
**Alcohol intake [*****n*** **(%)]**					
Never	69 (94.5)	159 (85.9)	76 (87.4)	19 (59.4)	<0.001
Occasionally	4 (5.5)	24 (13.0)	11 (12.6)	12 (37.5)	
Every day	0 (0.0)	2 (1.1)	0 (0.0)	1 (3.1)	
**Fresh fruit [*****n*** **(%)]**					
Never	23 (31.5)	51 (27.6)	17 (19.5)	4 (12.5)	0.071
Occasionally	36 (49.3)	82 (44.3)	42 (48.3)	13 (40.6)	
Every day	14 (19.2)	52 (28.1)	28 (32.2)	15 (46.9)	
**Milk [*****n*** **(%)]**					
Never	56 (76.7)	137 (74.1)	65 (74.7)	24 (75.0)	0.356
Occasionally	15 (20.5)	32 (17.3)	15 (17.2)	3 (9.4)	
Every day	2 (2.7)	16 (8.6)	7 (8.0)	5 (15.6)	
**Egg [*****n*** **(%)]**					
Never	16 (21.9)	48 (25.9)	22 (25.3)	6 (18.8)	0.028
Occasionally	41 (56.2)	90 (48.6)	31 (35.6)	11 (34.4)	
Every day	16 (21.9)	47 (25.4)	34 (39.1)	15 (46.9)	
**Chocolate [*****n*** **(%)]**					
Never	58 (79.5)	146 (78.9)	63 (72.4)	19 (59.4)	0.09
Occasionally	15 (20.5)	39 (21.1)	23 (26.4)	13 (40.6)	
Every day	0 (0.0)	0 (0.0)	1 (1.1)	0 (0.0)	
**Percentage of outdoor time [*****n*** **(%)]**					
<25%	26 (63.4)	70 (60.3)	50 (73.5)	22 (84.6)	0.166
25%−50%	11 (26.8)	39 (33.6)	15 (22.1)	4 (15.4)	
>50%	4 (9.8)	7 (6.0)	3 (4.4)	0 (0.0)	

The percentage of UC patients who consumed chocolate was highest in the eastern regions of China and lowest in the western regions (Table 6, *P* = 0.016).

**Table 6:** The difference in diet among ulcerative colitis patients from different regions in China.

	**Eastern regions *N* = 83**	**Middle regions *N* = 41**	**Western regions *N* = 62**	***P-*value**
**Alcohol intake [*****n*** **(%)]**				
Never	70 (84.3)	36 (87.8)	59 (95.2)	0.104
Occasionally	13 (15.7)	5 (12.2)	2 (3.2)	
Every day	0 (0.0)	0 (0.0)	1 (1.6)	
**Fresh fruit [*****n*** **(%)]**				
Never	19 (22.9)	11 (26.8)	12 (19.4)	0.746
Occasionally	43 (51.8)	17 (41.5)	30 (48.4)	
Every day	21 (25.3)	13 (31.7)	20 (32.3)	
**Milk [*****n*** **(%)]**				
Never	63 (75.9)	25 (61.0)	53 (85.5)	0.044
Occasionally	15 (18.1)	13 (31.7)	5 (8.1)	
Every day	5 (6.0)	3 (7.3)	4 (6.5)	
**Egg [*****n*** **(%)]**				
Never	15 (18.1)	6 (14.6)	12 (19.4)	0.802
Occasionally	35 (42.2)	14 (34.1)	24 (38.7)	
Every day	33 (39.8)	21 (51.2)	26 (41.9)	
**Chocolate [*****n*** **(%)]**				
Never	50 (60.2)	32 (78.0)	50 (80.6)	0.016
Occasionally	33 (39.8)	9 (22.0)	12 (19.4)	
Every day	–	–	–	

A higher percentage of UC patients from urban regions consumed alcohol compared to those from suburban regions, while fewer of them consumed alcohol every day (Table 7, *P* = 0.07). Additionally, UC patients from urban regions had a higher percentage of daily consumption of fresh fruit and chocolate intake (*P* = 0.047, *P* = 0.002, respectively) compared to suburban patients.

**Table 7:** The difference in diet between ulcerative colitis patients from suburban and urban regions in China.

	**Suburban regions *N* = 107**	**Urban regions *N* = 79**	***P-*value**
**Alcohol intake [*****n*** **(%)]**			
Never	99 (92.5)	66 (83.5)	0.070
Occasionally	7 (6.5)	13 (16.5)	
Every day	1 (0.9)	0 (0.0)	
**Fresh fruit [*****n*** **(%)]**			
Never	30 (28.0)	12 (15.2)	0.047
Occasionally	52 (48.6)	38 (48.1)	
Every day	25 (23.4)	29 (36.7)	
**Milk [*****n*** **(%)]**			
Never	80 (74.8)	61 (77.2)	0.664
Occasionally	21 (19.6)	12 (15.2)	
Every day	6 (5.6)	6 (7.6)	
**Egg [*****n*** **(%)]**			
Never	18 (16.8)	15 (19.0)	0.659
Occasionally	45 (42.1)	28 (35.4)	
Every day	44 (41.1)	36 (45.6)	
**Chocolate [*****n*** **(%)]**			
Never	86 (80.4)	46 (58.2)	0.002
Occasionally	21 (19.6)	33 (41.8)	
Every day	–	–	

No significant differences were observed in diet between UC patients with different levels of per capita monthly income (Table 8).

**Table 8:** The difference in diet in ulcerative colitis patients with different levels of per capita monthly income in China.

	** <2,000 yuan *N* = 25**	**2,000–5,000 yuan *N* = 92**	**5,000−10,000 yuan *N* = 48**	**>10,000 yuan *N* = 21**	***P*-value**
**Alcohol intake [*****n*** **(%)]**					
Never	22 (88.0)	86 (93.5)	39 (81.2)	18 (85.7)	0.066
Occasionally	2 (8.0)	6 (6.5)	9 (18.8)	3 (14.3)	
Every day	1 (4.0)	0 (0.0)	0 (0.0)	0 (0.0)	
**Fresh fruit [*****n*** **(%)]**					
Never	8 (32.0)	18 (19.6)	14 (29.2)	2 (9.5)	0.253
Occasionally	12 (48.0)	46 (50.0)	18 (37.5)	14 (66.7)	
Every day	5 (20.0)	28 (30.4)	16 (33.3)	5 (23.8)	
**Milk [*****n*** **(%)]**					
Never	21 (84.0)	71 (77.2)	34 (70.8)	15 (71.4)	0.797
Occasionally	2 (8.0)	16 (17.4)	11 (22.9)	4 (19.0)	
Every day	2 (8.0)	5 (5.4)	3 (6.2)	2 (9.5)	
**Egg [*****n*** **(%)]**					
Never	4 (16.0)	14 (15.2)	11 (22.9)	4 (19.0)	0.787
Occasionally	12 (48.0)	37 (40.2)	18 (37.5)	6 (28.6)	
Every day	9 (36.0)	41 (44.6)	19 (39.6)	11 (52.4)	
**Chocolate [*****n*** **(%)]**					
Never	20 (80.0)	68 (73.9)	31 (64.6)	13 (61.9)	0.362
Occasionally	5 (20.0)	24 (26.1)	17 (35.4)	8 (38.1)	
Every day	–	–	–		

## 4 Discussion

Within this multicenter case-control study, we found that appropriate consumption levels of alcohol, milk, and fresh fruit may reduce the risk of both CD and UC. Additionally, outdoor time of more than 25% of the day was identified as a protective factor for CD, while the intake of eggs and chocolate were risk factors for both CD and UC. Specifically, our analysis revealed higher egg consumption among CD patients in eastern regions of China and higher chocolate consumption among UC patients in the same region. Moreover, CD patients in these regions had lower levels of outdoor time, indicating a potential risk for IBD onset. Furthermore, IBD patients from urban regions or with higher per capita monthly income demonstrated increased consumption of fruit, eggs, and chocolate. These findings provide valuable evidence for developing region-specific prevention strategies for IBD.

Fresh fruit consumption was found to be a protective factor for both CD and UC in our research. Previous studies have shown varying results regarding the association between fruit intake and IBD risk. Some studies indicated that high fruit intake was associated with a decreased risk of CD (20, 21), while the relationship with UC remained inconclusive. A systematic review concluded that high fruit intake was associated with a decreased risk of CD but did not show a statistically significant association with UC (22). However, other studies by Li et al. (20) and Owczarek et al. (21) suggested that fruit intake was a protective factor for both CD and UC. Fruits are rich in fiber and vitamins, which are thought to have a protective effect against IBD risk (21). Pectin, a common fiber found in fresh fruits, can be metabolized by colon bacteria into short-chain fatty acids (SCFAs) with anti-inflammatory effects (23). Studies conducted on mouse models have observed that pectin directly inhibits the pro-inflammatory cytokine toll-like receptor (TLR) 1&2 pathways and downregulates inflammatory responses in the colon (24, 25). Further research could focus on investigating the specific amount and components of fruit that may play potential protective roles in the onset of IBD.

Our study found that different protein sources may have different effects on the onset of IBD. Egg consumption was found to be a risk factor, while moderate milk intake was protective for both CD and UC onset. However, fresh fish and red meat were not significantly associated with IBD onset. Previous studies have reported that high protein intake is associated with an increased risk of IBD (26) while the effect of different food rich in animal protein remains controversial. Jantchou et al. (26)found an association between both fish and meat consumption and the emergence of IBD, while egg and dairy consumption did not show such an association (26). Another study by Sakamoto et al. found that fish consumption was associated with an increased risk of CD but had no effect on UC (27). The potential mechanism why high protein diet increased the risk of IBD may be that it can increase microbial density, reduce microbial diversity and decrease intestinal barrier function, and was proved to increase the severity of colitis in mice (28). But the kind and amount of protein contained in each food varied, which may lead to the different effect on IBD onset. Red meat and eggs contain sulfur-containing amino acids, which can have negative effects on colon cells by increasing hydrogen sulfide concentration in the intestines and may contribute to relapse in UC patients (21, 29). This may explain why eggs were identified as a risk factor for IBD. On the other hand, the lack of association between red meat consumption and IBD onset in our study may be attributed to the relatively lower consumption of red meat among the Chinese population, resulting in insufficient power to trigger IBD emergence. Fish, apart from being a good source of protein, also contains high levels of omega-3 fatty acids, which have anti-inflammatory properties in various inflammatory diseases (30). A prospective study of 170,805 women suggested that greater intake of long-chain omega-3 fatty acids had a trend toward decreased UC risk (31). However, due to the complex composition and species variability of fish, it is challenging to draw a clear conclusion regarding its effect on IBD onset. Milk protein composition is also complex and may have implications for immune-related diseases. Bioactive constituents of milk such as lactoferrin and milk extracellular vesicles have been reported to play an important role in maintaining intestinal health and are even being explored as potential therapeutic targets for enteritis (32, 33). In our study, we assessed food intake by frequency, but the methods used previously to measure food amounts varied across different studies. Therefore, the specific amount of protein intake could not be determined precisely, which may contribute to differences in the results reported. Ultimately, it is important to note that a Western-style high protein diet is not recommended, and a balanced diet incorporating various food groups may help reduce the risk of IBD. For future studies, more precise methods for measuring food amounts and specific protein content should be employed to further explore the association between diet and IBD onset.

In our study, we found that alcohol consumption was protective against the onset of both CD and UC, while cigarette smoking did not show a significant effect on either CD or UC. The effect of alcohol on IBD development remains unclear, as previous studies have reported conflicting findings. A case-control study conducted in Japan included 384 UC patients and 384 healthy controls and found a protective effect of alcohol consumption on UC onset in 1994, but this effect was negated when alcohol consumption was combined with smoking (34). Furthermore, Miyake et al. (35) found that the influence of alcohol consumption on UC onset may depend on specific gene backgrounds among 384 UC cases and 661 healthy controls in Japan. On the other hand, a prospective cohort study including 198 incident UC cases, 792 controls, 84 CD cases, and 336 controls from six European countries reported no association between alcohol use and the onset of IBD (36). No significant differences were observed in CD development between individuals with and without alcohol consumption (37). The potential beneficial effect of alcohol consumption on IBD may be attributed to the presence of phenols in alcoholic beverages, which are rich in antioxidants and have inhibitory effects on the production and function of pro-inflammatory cytokines (38). However, excessive intake of ethanol can lead to the overproduction of acetaldehyde, which is toxic to the gut (39). Few studies have examined the association between alcohol and IBD onset, as most research tends to focus on the dietary habits of individuals diagnosed with IBD already, with alcohol generally not recommended for IBD patients. Regarding cigarette smoking, researchers tend to consider it as being associated with a higher risk of CD (40), while its effect on UC onset still requires further exploration. In our study, we observed a trend suggesting that smoking may be associated with a higher risk of CD, although the effect did not reach statistical significance. Previous studies have also reported that factors such as gender, ethnic background, and exposure to passive smoking in non-smokers may influence the results (41). However, we did not specifically capture information on passive smoking experience among participants, which could potentially affect the results. Further research is needed to explore the mechanisms through which smoking affects the onset of IBD.

In our study, we found that chocolate consumption was associated with an increased risk of IBD onset. Excessive consumption of dietary sugar is widely accepted as being associated with a higher risk of IBD. Specifically, the consumption of sugar-sweetened beverages and chocolate has been highlighted as factors contributing to the increased incidence of IBD (42), which is consistent with the findings of our study. Chocolate, as a representative example of Western-style food, is known for its high calorie content, particularly from sugar. It is considered an ultra-processed food and is generally not recommended for excessive consumption due to its potential negative health effects.

Vitamin D deficiency is a common phenomenon observed in IBD patients, and some researchers consider it as a potential factor that may increase the risk of developing IBD (21). In our study, we found that outdoor time of more than 25% of the day was protective against CD onset, likely due to increased levels of sunlight exposure and synthesis of vitamin D. One of the most significant non-skeletal benefits of vitamin D is its activity as an immuno-modulator with potent anti-inflammatory effects, which may play an important role in maintenance of gastrointestinal barrier integrity and regulating intestinal permeability, potentially protecting against the development and progression of IBD (43). In mouse models, the active form of vitamin D, known as 1,25-dihydroxyvitamin D (1,25-(OH)2D), has been shown to regulate the function of the gastrointestinal microbiota and promote anti-inflammatory and tolerogenic immune responses (44). Therefore, ensuring sufficient outdoor time and sunlight exposure or considering vitamin D supplementation may be helpful in reducing the risk of CD.

The association between different surgeries and IBD remained inconsistent. A previous study conducted by Koutroubakis et al. (13) found that both appendectomy and tonsillectomy were identified as risk factors for CD. Lee et al. (45) also observed an increased risk of both CD and UC after appendectomy, while some other studies suggested an inverse relationship between appendectomy and UC and even showed previous appendectomy may attenuate the severity of UC (46–48). A systematic review and meta-analysis published by Sun et al. (49) demonstrated the association between an increased risk of developing CD and tonsillectomy though no relationship was found between tonsillectomy and UC. The underlying mechanism remained unclear and was suspected owing to the potential immunological functions of appendix and tonsil, and the absence of appendix or tonsil could influence the immune response in gut and the composition of gut microbiota (45, 49, 50). On the other hand, Zhong et al. and Wang et al. suggested that Helicobacter pylori (Hp) infection was a protective factor for IBD, and eradication of Hp could lead to the recurrence of IBD (51, 52). The mechanism was probable to be the downregulation of pro-inflammatory responses and inflammatory pathways, as well as altered composition of the large intestine microbiota (51). But we didn't find the association between IBD and appendectomy, tonsillectomy or Hp infection history in multivariate analysis, which was possibly because the difference in population included and other environment factors between our research and the previous research.

In a microscopic perspective, diet may have an influence on IBD onset and disease activity owing to the alteration of gut microbiome (53, 54). Normal gut microbiome plays a pivotal role in maintaining overall health, and influences various physiological processes including digestion, metabolism, absorption of nutrients, regulation of immune system, as well as providing a gut barrier function (53). Normal gut microbiome consists of four major bacterial phyla, that is Firmicutes, Bacteroidetes, Proteobacteria, and Actinobacteria (53). In population with different levels of urbanization and different diet patterns, abundance of those four major gut bacterial phyla differs (8), which may furtherly result in the risk of IBD emergence. Previous studies have also demonstrated the reduced abundance of the phylum Firmicutes and an increase of the phylum Proteobacteria in IBD patients (54). But the relationship between diet, gut microbiome and IBD remains less clear and is needed to be furtherly explored.

The incidence of IBD in China has been reported to vary considerably, with the highest rates observed in regions characterized by greater urbanization and economic advancement (55, 56). In light of this, we further analyzed the differences in dietary patterns among IBD patients from different regions in China or with different levels of per capita monthly income. Our focus primarily centered on the risky and protective foods mentioned earlier, with the aim of identifying any tendencies or preferences within the Chinese population. Our findings revealed that CD patients from eastern regions of China tended to consume more eggs while having less outdoor time, and UC patients in this region consumed more chocolate, all of which are considered risk factors for IBD. A study investigating the trend and geographic variation of IBD incidence and prevalence in China also noted an east-to-west gradient, with a greater burden observed in the eastern region (6). This may be associated with inappropriate dietary choices. Among CD patients from urban regions or with higher per capita monthly income, we found that they had greater freedom in their food choices, consuming more fruits but also more eggs and chocolate, while having a lower proportion of outdoor time. For these populations, who have better economic conditions and are located in urban areas, we recommend adopting a balanced diet that includes an increased intake of fresh fruit while ensuring appropriate consumption of alcohol, animal protein, and milk. It is important to moderate the consumption of sugar-sweetened beverages and chocolate.

### 4.1 Strengths and limitations

The primary strength of this study lies in the various methods used to control bias. Firstly, the consecutive enrollment of research participants helped minimize selection bias. Secondly, an electronic questionnaire survey was employed with carefully designed questions to ensure clarity and reduce information bias. Thirdly, healthy controls were relatives of IBD patients or individuals from the same regions, which helped control for genetic confounders and other non-dietary biases within the control group. Additionally, propensity score matching was implemented to further manage potential confounding biases. Lastly, multivariate analysis was conducted to identify the most associated factors related to IBD onset. However, there are limitations to consider in this study. As a case-control study, it can only indicate correlations between the explored factors and IBD, but it cannot establish causal relationships. Altered gut microbiome may be a potential cause of IBD and is probably to be related to a changing diet pattern, so future research could focus on exploring the causal relationship of changing diet, gut microbiome and IBD emergence. Furthermore, the included patients were at different phases of the disease, and those with recurrent symptoms may have experienced recall bias when reporting their pre-disease state and diet. Lastly, a more precise method to measure food quantities was not utilized, and outdoor time could only partially show the amount of sunlight exposure, which could be addressed in future studies. Therefore, future research could focus on specific types of food that are easier to measure in terms of quantity and specify the nutritional constituents contained in each food item, and the reasons why outdoor time have an association with risk of CD should be furtherly explored.

## 5 Conclusion

This study aimed to investigate the association between diet, outdoor time, and the onset of IBD. In conclusion, we found that moderate consumption of alcohol and milk, daily consumption of fresh fruit, and outdoor time of more than 25% of the day were protective factors for CD onset. Additionally, alcohol consumption, milk intake, and daily consumption of fresh fruit showed protective effects against UC onset. On the other hand, egg and chocolate consumption were identified as risk factors for both CD and UC. These findings underscore the importance of maintaining a balanced diet, particularly among populations from the eastern regions of China, urban areas, and those with higher levels of per capita monthly income. Future research will focus on exploring appropriate food quantities and specific food types within a well-balanced and healthy diet.

## Data availability statement

The raw data supporting the conclusions of this article will be made available by the authors, without undue reservation.

## Ethics statement

The studies involving humans were approved by the Peking Union Medical College Hospital and the Chinese Academy Medical Science Ethics Committee. The studies were conducted in accordance with the local legislation and institutional requirements. The participants provided their written informed consent to participate in this study.

## Author contributions

XChu: Writing – review & editing, Data curation, Formal analysis, Investigation, Writing – original draft. XChe: Investigation, Writing – original draft, Writing – review & editing, Methodology. HZ: Investigation, Writing – review & editing. YW: Writing – review & editing, Data curation, Validation. HG: Data curation, Writing – review & editing. YC: Writing – review & editing, Formal analysis. XL: Writing – review & editing, Data curation. ZZ: Data curation, Writing – review & editing. YH: Data curation, Writing – review & editing. XD: Data curation, Writing – review & editing. QW: Data curation, Writing – review & editing. CZ: Data curation, Writing – review & editing. XC: Data curation, Writing – review & editing. HY: Writing – review & editing, Conceptualization, Funding acquisition, Methodology, Validation. JQ: Conceptualization, Methodology, Validation, Writing – review & editing.
