# Supplementary material for: Association of diet and outdoor time with inflammatory bowel disease: a multicenter case-control study using propensity matching analysis in China
Source: Front Public Health. 2024 Jun 17;12:1368401. doi: 10.3389/fpubh.2024.1368401 (PMC11215971; doi:10.3389/fpubh.2024.1368401)
Supplement: Supplementary file 1 [file Table_1.DOCX]

**Supplementary Table 1. Distribution of IBD patients and healthy controls across China**

| Regions of China | CD patients | HC matched with CD patients | UC patients | HC matched with UC patients |
| --- | --- | --- | --- | --- |
| Northeast China | 48(15.29) | 34(10.83) | 21(12.80) | 20(12.20) |
| North China | 43(13.69) | 74(23.57) | 49(29.88) | 45(27.44) |
| Northwest China | 7(2.23) | 21(6.69) | 8(4.88) | 4(2.44) |
| Central China | 49(15.61) | 49(15.61) | 13(7.93) | 29(17.68) |
| East China | 101(32.17) | 86(27.39) | 26(15.85) | 40(24.39) |
| Southwest China | 55(17.52) | 45(14.33) | 43(26.22) | 23(14.02) |
| South China | 11(3.50) | 5)1.59) | 4(2.44) | 3(1.83) |
| Total | 314 | 314 | 164 | 164 |

Abbreviations:

IBD: inflammatory bowel disease; CD: Crohn’s disease; UC: ulcerative colitis.
