# Supplementary material for: Association of diet and outdoor time with inflammatory bowel disease: a multicenter case-control study using propensity matching analysis in China
Source: Front Public Health. 2024 Jun 17;12:1368401. doi: 10.3389/fpubh.2024.1368401 (PMC11215971; doi:10.3389/fpubh.2024.1368401)
Supplement: Supplementary file 2 [file Table_2.DOCX]

Supplementary Table 2

Diet Questionnaire:

| Participant information | | |
| --- | --- | --- |
| Q1 | What is your name (Given name/Surname)? | |
| Q2 | If you are an IBD patient, what is your diagnosis？   - Crohn’s disease - Ulcerative colitis - Inflammatory bowel disease unclassified (IBDU) | |
| Q3 | What is your gender？   - Male; - Female; | |
| Q4 | How old are you? | |
| Q5 | What is your blood types?   - A; - B; - O; - AB; | |
| Q6 | What is your nationality?   - Hans; - Others; | |
| Q7 | Do you have a family history of IBD?   - Yes; Who____________ - No; | |
| Questions about diets | | |
| Q8 | | How often do you consume fresh vegetables?   - Every day； - Occasionally (once a week to once a month)； - Never； |
| Q9 | | How often do you consume fresh fruit?   - Every day； - Occasionally (once a week to once a month)； - Never； |
| Q10 | | How often do you consume red meat ?   - Every day； - Occasionally (once a week to once a month)； - Never； |
| Q11 | | How often do you consume fresh fish?   - Every day； - Occasionally (once a week to once a month)； - Never； |
| Q12 | | How often do you consume salted fish?   - Every day； - Occasionally (once a week to once a month)； - Never； |
| Q13 | | How often do you consume shellfish?   - Every day； - Occasionally (once a week to once a month)； - Never； |
| Q14 | | How often do you consume crabs?   - Every day； - Occasionally (once a week to once a month)； - Never； |
| Q15 | | How often do you consume shrimps?   - Every day； - Occasionally (once a week to once a month)； - Never； |
| Q16 | | How often do you consume milk?   - Every day； - Occasionally (once a week to once a month)； - Never； |
| Q17 | | How often do you consume yogurt?   - Every day； - Occasionally (once a week to once a month)； - Never； |
| Q18 | | How often do you consume eggs?   - Every day； - Occasionally (once a week to once a month)； - Never； |
| Q19 | | How often do you consume western-style fast food ?   - Every day； - Occasionally (once a week to once a month)； - Never； |
| Q20 | | How often do you consume fried food?   - Every day； - Occasionally (once a week to once a month)； - Never； |
| Q21 | | How often do you consume coarse grain?   - Every day； - Occasionally (once a week to once a month)； - Never； |
| Q22 | | How often do you consume tea?   - Every day； - Occasionally (once a week to once a month)； - Never; |
| Q23 | | How often do you consume coffee?   - Every day； - Occasionally (once a week to once a month)； - Never； |
| Q24 | | How often do you consume food stored in refrigerator more than 3 days?   - Every day； - Occasionally (once a week to once a month)； - Never； |
| Q25 | | How often do you consume raw seafood?   - Every day； - Occasionally (once a week to once a month)； - Never； |
| Q26 | | How often do you consume raw vegetables?   - Every day； - Occasionally (once a week to once a month)； - Never； |
| Q27 | | How often do you consume chocolates?   - Every day； - Occasionally (once a week to once a month)； - Never； |
| Questions about habits | | |
| Q28 | | Have you ever smoked?   - Never; - Ever (including people smoke at present); |
| Q29 | | How often do you consume alcohol?   - Every day； - Occasionally (once a week to once a month)； - Never； |
| Q30 | | How long do you spend outdoors in average every day?   - ＜25% of a day; - 25-50% of a day; - >50% of a day； |
| Q31 | | Please select all the surgery or disease you have had before 20 years old and IBD onset?   - Appendectomy; - Tonsillectomy; - Cholecystectomy; - Asthma; - Eczema; - Helicobacter pylori (HP) infection. |
