# Supplementary material for: Association of diet and outdoor time with inflammatory bowel disease: a multicenter case-control study using propensity matching analysis in China
Source: Front Public Health. 2024 Jun 17;12:1368401. doi: 10.3389/fpubh.2024.1368401 (PMC11215971; doi:10.3389/fpubh.2024.1368401)
Supplement: Supplementary file 3 [file Table_3.DOCX]

| Supplementary Table 3. Characteristics of CD patients and HC before and after PSM. | | | | | | |  |  |
| --- | --- | --- | --- | --- | --- | --- | --- | --- |
| Characteristic | HC before PSM  (n=667) | CD before PSM  (n=377) | *P* value | *SMD* | HC after PSM  (n=314) | CD after PSM  (n=314) | *P* value | *SMD* |
| Age [median ( IQR)] | 34.00 [29.00, 43.00] | 35.00 [30.00, 43.00] | 0.646 | 0.016 | 35.00 [29.25, 44.75] | 34.00 [29.00, 41.75] | 0.133 | 0.114 |
| Gender |  |  | <0.001 | 0.39 |  |  | 1 | 0.007 |
| Male | 350 (52.5) | 268 (71.1) |  |  | 212 (67.5) | 213 (67.8) |  |  |
| Female | 317 (47.5) | 109 (28.9) |  |  | 102 (32.5) | 101 (32.2) |  |  |
| Ethnic group [n (%)] |  |  | 0.818 | 0.025 |  |  | 0.787 | 0.043 |
| Hans | 642 (96.3) | 361 (95.8) |  |  | 308 (98.1) | 306 (97.5) |  |  |
| Minority | 25 (3.7) | 16 (4.2) |  |  | 6 (1.9) | 8 (2.5) |  |  |
| Family history of IBD [n (%)] | 42 (6.3) | 4 (1.1) | <0.001 | 0.281 | 1 (0.3) | 1 (0.3) | 1 | <0.001 |
| University or higher education level [n (%)] | 500 (75.0) | 200 (53.1) | <0.001 | 0.469 | 197 (62.7) | 198 (63.1) | 1 | 0.007 |
| Birthplace [n (%)] |  |  |  |  |  |  | 1 | 0.006 |
| Urban | 356 (53.4) | 159 (42.2) | 0.001 | 0.226 | 147 (46.8) | 148 (47.1) |  |  |
| Suburban | 311 (46.6) | 218 (57.8) |  |  | 167 (53.2) | 166 (52.9) |  |  |
| Abbreviations:  CD: Crohn’s disease; PSM: propensity-score matching; IQR: interquartile range; SMD: standard mean difference. | | | | | | | | |
