# Supplementary material for: Association of diet and outdoor time with inflammatory bowel disease: a multicenter case-control study using propensity matching analysis in China
Source: Front Public Health. 2024 Jun 17;12:1368401. doi: 10.3389/fpubh.2024.1368401 (PMC11215971; doi:10.3389/fpubh.2024.1368401)
Supplement: Supplementary file 4 [file Table_4.DOCX]

| Supplementary Table 4. Characteristics of UC patients and HC before and after PSM. | | | | | | |  |  |
| --- | --- | --- | --- | --- | --- | --- | --- | --- |
| Characteristic | HC before PSM  (n=667) | UC before PSM  (n=186) | *P* value | *SMD* | HC after PSM  (n=164) | UC after PSM  (n=164) | *P* value | *SMD* |
| Age [median ( IQR)] | 34.00 [29.00, 43.00] | 40.00 [33.00, 51.00] | <0.001 | 0.475 | 40.00 [32.75, 50.00] | 39.00 [33.00, 49.00] | 0.901 | 0.016 |
| Gender |  |  | 0.52 | 0.06 |  |  | 1 | <0.001 |
| Male | 350 (52.5) | 92 (49.5) |  |  | 83 (50.6) | 83 (50.6) |  |  |
| Female | 317 (47.5) | 94 (50.5) |  |  | 81 (49.4) | 81 (49.4) |  |  |
| Ethnic group [n (%)] |  |  | 0.274 | 0.101 |  |  | 1 | <0.001 |
| Hans | 642 (96.3) | 175 (94.1) |  |  | 156 (95.1) | 156 (95.1) |  |  |
| Minority | 25 (3.7) | 11 (5.9) |  |  | 8 (4.9) | 8 (4.9) |  |  |
| Family history of IBD [n (%)] | 42 (6.3) | 10 (5.4) | 0.771 | 0.039 | 8 (4.9) | 7 (4.3) | 1 | 0.029 |
| University or higher education level [n (%)] | 500 (75.0) | 101 (54.3) | <0.001 | 0.443 | 98 (59.8) | 98 (59.8) | 1 | <0.001 |
| Birthplace [n (%)] |  |  | 0.011 | 0.22 |  |  | 1 | 0.012 |
| Urban | 356 (53.4) | 79 (42.5) |  |  | 70 (42.7) | 69 (42.1) |  |  |
| Suburban | 311 (46.6) | 107 (57.5) |  |  | 94 (57.3) | 95 (57.9) |  |  |
| Abbreviations:  UC: ulcerative colitis; PSM: propensity-score matching; IQR: interquartile range; SMD: standard mean difference. | | | | | | | | |
