# Supplementary material for: Association of diet and outdoor time with inflammatory bowel disease: a multicenter case-control study using propensity matching analysis in China
Source: Front Public Health. 2024 Jun 17;12:1368401. doi: 10.3389/fpubh.2024.1368401 (PMC11215971; doi:10.3389/fpubh.2024.1368401)
Supplement: Supplementary file 5 [file Table_5.DOCX]

| Supplementary Table 5. Differences of diet, smoking, alcohol intake and past medical history between IBD patients and HC | | | | | | |
| --- | --- | --- | --- | --- | --- | --- |
|  | HC after PSM  (n=314) | CD after PSM  (n=314) | *P* value | HC after PSM  (n=164) | UC after PSM  (n=164) | *P* value |
| Tonsillectomy history [n (%)] | 5 (1.6) | 6 (1.9) | 1 | 3 (1.8) | 0 (0.0) | 0.248 |
| Appendicectomy history [n (%)] | 11 (3.5) | 13 (4.1) | 0.835 | 7 (4.3) | 2 (1.2) | 0.176 |
| Cholecystectomy history [n (%)] | 1 (0.3) | 2 (0.6) | 1 | 1 (0.6) | 0 (0.0) | 1 |
| Asthma history [n (%)] | 7 (2.2) | 6 (1.9) | 0.484 | 4 (2.4) | 5 (3.0) | 1 |
| Eczema history [n (%)] | 29 (9.2) | 35 (11.1) | 0.658 | 14 (8.5) | 23 (14.0) | 1 |
| Hp infection history [n (%)] |  |  | 0.026* |  |  | <0.001* |
| No | 187 (59.6) | 162 (51.6) |  | 99 (60.4) | 79 (48.2) |  |
| Yes | 34 (10.8) | 27 (8.6) |  | 29 (17.7) | 16 (9.8) |  |
| Unclear | 93 (29.6) | 125 (39.8) |  | 36 (22.0) | 69 (42.1) |  |
| Smoking [n (%)] |  |  | 0.645 |  |  | 0.213 |
| Never | 238 (75.8) | 233 (74.2) |  | 136 (82.9) | 127 (77.4) |  |
| Ever | 76(24.2) | 81(25.8) |  | 28 (17.1) | 37 (22.6) |  |
| Alcohol intake [n (%)] |  |  | <0.001* |  |  | <0.001* |
| Never | 182 (58.0) | 270 (86.0) |  | 105 (64.0) | 145 (88.4) |  |
| Occasionally | 115 (36.6) | 42 (13.4) |  | 50 (30.5) | 18 (11.0) |  |
| Every day | 17 (5.4) | 2 (0.6) |  | 9 (5.5) | 1 (0.6) |  |
| Fresh vegetables [n (%)] |  |  | 0.795 |  |  | 0.935 |
| Never | 39 (12.4) | 43 (13.7) |  | 21 (12.8) | 21 (12.8) |  |
| Occasionally | 84 (26.8) | 88 (28.0) |  | 53 (32.3) | 56 (34.1) |  |
| Every day | 191 (60.8) | 183 (58.3) |  | 90 (54.9) | 87 (53.0) |  |
| Fresh fruit [n (%)] |  |  | 0.001* |  |  | 0.007* |
| Never | 62 (19.7) | 79 (25.2) |  | 31 (18.9) | 37 (22.6) |  |
| Occasionally | 111 (35.4) | 139 (44.3) |  | 64 (39.0) | 85 (51.8) |  |
| Every day | 141 (44.9) | 96 (30.6) |  | 69 (42.1) | 42 (25.6) |  |
| Red meat [n (%)] |  |  | 0.005* |  |  | 0.566 |
| Never | 75 (23.9) | 47 (15.0) |  | 33 (20.1) | 26 (15.9) |  |
| Occasionally | 80 (25.5) | 108 (34.4) |  | 57 (34.8) | 63 (38.4) |  |
| Every day | 159 (50.6) | 159 (50.6) |  | 74 (45.1) | 75 (45.7) |  |
| Fresh fish [n (%)] |  |  | 0.001* |  |  | 0.112 |
| Never | 140 (44.6) | 95 (30.3) |  | 68 (41.5) | 58 (35.4) |  |
| Occasionally | 150 (47.8) | 182 (58.0) |  | 80 (48.8) | 97 (59.1) |  |
| Every day | 24 (7.6) | 37 (11.8) |  | 16 (9.8) | 9 (5.5) |  |
| Salted fish [n (%)] |  |  | 0.441 |  |  | 1 |
| Never | 268 (85.4) | 258 (82.2) |  | 137 (83.5) | 136 (82.9) |  |
| Occasionally | 43 (13.7) | 54 (17.2) |  | 25 (15.2) | 26 (15.9) |  |
| Every day | 3 (1.0) | 2 (0.6) |  | 2 (1.2) | 2 (1.2) |  |
| Shellfish[n (%)] |  |  | 1 |  |  | 0.063 |
| Never | 265 (84.4) | 266 (84.7) |  | 138 (84.1) | 150 (91.5) |  |
| Occasionally | 49 (15.6) | 48 (15.3) |  | 26 (15.9) | 14 (8.5) |  |
| Every day | 0 (0) | 0 (0) |  | 0 (0) | 0 (0) |  |
| Crab [n (%)] |  |  | 0.751 |  |  | 0.239 |
| Never | 261 (83.1) | 258 (82.2) |  | 139 (84.8) | 148 (90.2) |  |
| Occasionally | 52 (16.6) | 56 (17.8) |  | 24 (14.6) | 15 (9.1) |  |
| Every day | 1 (0.3) | 0 (0.0) |  | 1 (0.6) | 1 (0.6) |  |
| Shrimp [n (%)] |  |  | 0.434 |  |  | 0.728 |
| Never | 224 (71.3) | 216 (68.8) |  | 114 (69.5) | 120 (73.2) |  |
| Occasionally | 87 (27.7) | 97 (30.9) |  | 47 (28.7) | 42 (25.6) |  |
| Every day | 3 (1.0) | 1 (0.3) |  | 3 (1.8) | 2 (1.2) |  |
| Milk[n (%)] |  |  | <0.001* |  |  | 0.006* |
| Never | 182 (58.0) | 235 (74.8) |  | 97 (59.1) | 124 (75.6) |  |
| Occasionally | 88 (28.0) | 53 (16.9) |  | 49 (29.9) | 30 (18.3) |  |
| Every day | 44 (14.0) | 26 (8.3) |  | 18 (11.0) | 10 (6.1) |  |
| Yogurt [n (%)] |  |  | 0.115 |  |  | 0.492 |
| Never | 185 (58.9) | 168 (53.5) |  | 99 (60.4) | 90 (54.9) |  |
| Occasionally | 97 (30.9) | 121 (38.5) |  | 47 (28.7) | 57 (34.8) |  |
| Every day | 32 (10.2) | 25 (8.0) |  | 18 (11.0) | 17 (10.4) |  |
| Egg [n (%)] |  |  | 0.001* |  |  | 0.017* |
| Never | 114 (36.3) | 75 (23.9) |  | 53 (32.3) | 31 (18.9) |  |
| Occasionally | 105 (33.4) | 140 (44.6) |  | 59 (36.0) | 65 (39.6) |  |
| Every day | 95 (30.3) | 99 (31.5) |  | 52 (31.7) | 68 (41.5) |  |
| Western-style fast food [n (%)] |  |  | 0.373 |  |  | 0.62 |
| Never | 271 (86.3) | 262 (83.4) |  | 145 (88.4) | 141 (86.0) |  |
| Occasionally | 43 (13.7) | 52 (16.6) |  | 19 (11.6) | 23 (14.0) |  |
| Every day | 0 (0) | 0 (0) |  | 0 (0) | 0 (0) |  |
| Fried food [n (%)] |  |  | 0.217 |  |  | 0.709 |
| Never | 226 (72.0) | 207 (65.9) |  | 110 (67.1) | 107 (65.2) |  |
| Occasionally | 76 (24.2) | 89 (28.3) |  | 48 (29.3) | 53 (32.3) |  |
| Every day | 12 (3.8) | 18 (5.7) |  | 6 (3.7) | 4 (2.4) |  |
| Coarse grain [n (%)] |  |  | <0.001* |  |  | 0.472 |
| Never | 204 (65.0) | 244 (77.7) |  | 103 (62.8) | 113 (68.9) |  |
| Occasionally | 76 (24.2) | 59 (18.8) |  | 41 (25.0) | 36 (22.0) |  |
| Every day | 34 (10.8) | 11 (3.5) |  | 20 (12.2) | 15 (9.1) |  |
| Tea [n (%)] |  |  | 0.005* |  |  | 0.243 |
| Never | 221 (70.4) | 255 (81.2) |  | 128 (78.0) | 128 (78.0) |  |
| Occasionally | 50 (15.9) | 36 (11.5) |  | 18 (11.0) | 25 (15.2) |  |
| Every day | 43 (13.7) | 23 (7.3) |  | 18 (11.0) | 11 (6.7) |  |
| Coffee [n (%)] |  |  | 0.47 |  |  | 0.023* |
| Never | 271 (86.3) | 280 (89.2) |  | 143 (87.2) | 146 (89.0) |  |
| Occasionally | 36 (11.5) | 30 (9.6) |  | 14 (8.5) | 18 (11.0) |  |
| Every day | 7 (2.2) | 4 (1.3) |  | 7 (4.3) | 0 (0.0) |  |
| Food stored in refrigerator ≥ 3 days [n (%)] |  |  | 0.217 |  |  | 0.28 |
| Never | 226 (72.0) | 207 (65.9) |  | 106 (64.6) | 92 (56.1) |  |
| Occasionally | 76 (24.2) | 89 (28.3) |  | 53 (32.3) | 65 (39.6) |  |
| Every day | 12 (3.8) | 18 (5.7) |  | 5 (3.0) | 7 (4.3) |  |
| Raw seafood [n (%)] |  |  | 0.002* |  |  | 0.016* |
| Never | 263 (83.8) | 288 (91.7) |  | 132 (80.5) | 148 (90.2) |  |
| Occasionally | 47 (15.0) | 26 (8.3) |  | 29 (17.7) | 16 (9.8) |  |
| Every day | 4 (1.3) | 0 (0.0) |  | 3 (1.8) | 0 (0.0) |  |
| Raw vegetable [n (%)] |  |  | 0.008* |  |  | 0.049* |
| Never | 232 (73.9) | 255 (81.2) |  | 118 (72.0) | 129 (78.7) |  |
| Occasionally | 43 (13.7) | 42 (13.4) |  | 24 (14.6) | 26 (15.9) |  |
| Every day | 39 (12.4) | 17 (5.4) |  | 22 (13.4) | 9 (5.5) |  |
| Chocolate [n (%)] |  |  | 0.045* |  |  | 0.001* |
| Never | 255 (81.2) | 236 (75.2) |  | 139 (84.8) | 113 (68.9) |  |
| Occasionally | 55 (17.5) | 77 (24.5) |  | 24 (14.6) | 51 (31.1) |  |
| Every day | 4 (1.3) | 1 (0.3) |  | 1 (0.6) | 0 (0.0) |  |
| Percentage of outdoor time every day [n (%)] |  |  | 0.003* |  |  | 0.338 |
| <25% | 109 (57.1) | 145 (70.0) |  | 57 (57.0) | 69 (67.0) |  |
| 25-50% | 56 (29.3) | 52 (25.1) |  | 31 (31.0) | 25 (24.3) |  |
| >50% | 26 (13.6) | 10 (4.8) |  | 12 (12.0) | 9 (8.7) |  |
| Abbreviations:  CD: Crohn’s disease; UC: ulcerative colitis; PSM: propensity-score matching; Hp: helicobacter pylori. | | | | | | |
